# Association of the Swiss Diagnosis-Related Group Reimbursement System With Length of Stay, Mortality, and Readmission Rates in Hospitalized Adult Patients

**DOI:** 10.1001/jamanetworkopen.2018.8332

**Published:** 2019-02-15

**Authors:** Alexander Kutz, Lara Gut, Fahim Ebrahimi, Ulrich Wagner, Philipp Schuetz, Beat Mueller

**Affiliations:** 1 Division of General Internal and Emergency Medicine, University Department of Medicine, Kantonsspital Aarau, Aarau, Switzerland; 2 Division of Endocrinology, University Hospital Basel, Basel, Switzerland; 3 Division of Health and Social Affairs, Section Health, Swiss Federal Office for Statistics, Neuchâtel, Switzerland; 4 Faculty of Medicine, University of Basel, Basel, Switzerland

## Abstract

**Importance:**

In 2012, hospital reimbursement in Switzerland changed from a fee-for-service per diem system to a diagnosis-related group (SwissDRG) system. Whether this change in reimbursement is associated with harmful implications for quality of care and patient outcomes remains unclear.

**Objective:**

To examine the association of the SwissDRG implementation with length of hospital stay (LOS), in-hospital mortality, and 30-day readmission rates in the overall adult inpatient population and stratified by 5 individual diagnoses.

**Design, Setting, and Participants:**

This cohort study used administrative data from the Swiss Federal Statistical Office to investigate medical hospitalizations in Switzerland from January 1, 2009, through December 31, 2015. All hospitalizations for adult medical inpatients were included in the main analysis. Patients who presented with 1 of the 5 common medical diagnoses were included in the subanalyses: community-acquired pneumonia, exacerbation of chronic obstructive pulmonary disease, acute myocardial infarction, acute heart failure, and pulmonary embolism. An interrupted time series model was used to determine changes in time trends for risk-adjusted LOS, in-hospital mortality, and 30-day readmission after the implementation of SwissDRG in 2012. Analyses were performed from March 1, 2018, to June 30, 2018, and from November 1, 2018, to December 18, 2018.

**Main Outcomes and Measures:**

Monthly patient-level data for LOS, in-hospital mortality, and 30-day readmission rates.

**Results:**

The sample included a total of 2 426 722 hospitalized adult patients. Of this total, 1 018 404 patients (41.9%; 531 226 [52.2%] male, median [interquartile range (IQR)] age of 69 [55-80] years) composed the before-SwissDRG period; 1 408 318 patients (58.0%; 730 228 [51.9%] male, median [IQR] age of 70 [56-81] years) composed the after-SwissDRG period. The overall LOS gradually decreased from unadjusted mean (SD) 8.0 (12.7) days in 2009 to 7.2 (17.3) days in 2015. This reduction in LOS, however, was not substantially greater with the implementation of SwissDRG in 2012 (risk-adjusted slope, –0.0166 days; 95% CI, –0.0223 to –0.0110 days), with an adjusted difference in slopes of 0.0000 days (95% CI, –0.0072 to 0.0072 days). Risk-adjusted all-cause in-hospital mortality declined from 4.9% in 2009 to 4.6% in 2015, with a substantially greater decline after implementation of SwissDRG (difference between monthly slopes before and after implementation, –0.0115%; 95% CI, –0.0190% to –0.0039%). In the same period, risk-adjusted 30-day readmission rates increased from 14.4% in 2009 to 15.0% in 2015, with a greater increase after SwissDRG implementation (change in monthly slope, 0.0339%; 95% CI, 0.0254%-0.0423%). Patients with acute myocardial infarction were found to have a substantially greater increase after SwissDRG implementation in 30-day readmission rates (adjusted difference in slopes, 0.1144%; 95% CI, 0.0617%-0.1671%).

**Conclusions and Relevance:**

Among medical hospitalizations in Switzerland, SwissDRG implementation appeared to be associated with an increase in readmission rates and a decrease in in-hospital mortality but not with the gradual decrease in LOS observed in the historical control period.

## Key Points

**Question:**

Is the implementation of the Swiss diagnosis-related group reimbursement system associated with a reduction in length of hospital stay without negatively affecting in-hospital mortality and readmission rates in adult patients?

**Findings:**

In this cohort study of data from 2 426 722 adult patients, the gradual decrease in length of hospital stay observed from 2009 to 2015 was not substantially greater after the implementation of the Swiss diagnosis-related group system in 2012. An increase in 30-day readmission rates and a decrease in in-hospital mortality were observed after the introduction of the Swiss diagnosis-related group system.

**Meaning:**

Swiss diagnosis-related group implementation appeared to be associated with higher readmission rates and lower in-hospital mortality but not with a substantial decrease in length of hospital stays.

## Teaser

This cohort study examines hospitalization and patient outcomes data of hospitalized adult patients before and after the implementation of the diagnosis-related group reimbursement system in Switzerland.

## Introduction

In Switzerland, health care spending is among the highest in the world and constantly rising.^1^ As in other countries, the recent progress in medical treatment in Switzerland has reduced disease-related mortality and morbidity, which in turn augments the population of multimorbid patients with recurrent in-hospital treatments owing to acute decompensation of chronic illnesses. This situation reinforces the need in polymorbid patients to improve in-hospital treatments with a well-coordinated continuum of care, including social services and nursing care.^2^ Other concerns are overtreatment and overconsumption,^3^ as most services in Switzerland are covered by the mandatory health insurance.^4^

To address health care spending for in-hospital treatment, the Swiss parliament passed a law in 2007 to change reimbursement from a fee-for-service per diem system to a prospective payment system based on diagnosis-related group (SwissDRG), which was introduced in 2012 across Switzerland. However, some cantons had implemented the SwissDRG reimbursement a few years before 2012, mirroring a phase-in period in other countries. Like other DRG-based payment systems, the SwissDRG system reimburses hospitals for acute inpatient hospital services with a fixed rate per case. Each hospital admission is assigned to a case group (pool) on the basis of specific criteria shared by all individuals in the group (eg, the primary diagnosis, secondary diagnoses, treatments, and degree of severity); the pool is then provided with a cost weight.^5^ The remuneration is calculated by multiplying the cost weight of the SwissDRG with the base rate set by the local cantons. Unlike the fee-for-service per diem system, the SwissDRG system provides incentives for early discharge of patients, resulting in shorter length of hospital stay (LOS) and cost advantages. Still, the economic and clinical consequences associated with the introduction of SwissDRG remain unclear.^6^

There is concern that the introduction of SwissDRG would result in lower quality of care owing to inadequate and premature discharges of inpatients with increased risk for mortality or rehospitalization, as colloquially called *bloody* discharges.^7^ This potentially harmful trend has been found in other countries after the introduction of a DRG reimbursement system^8,9,10,11,12,13,14,15^ but showed mixed results in the United States and Germany.^14,16,17,18,19^ Better understanding of the association between the DRG reimbursement and quality of care is important to adapt in-hospital health care processes and promote data transparency, which influences the public and policymakers.

To better understand the health care implications of nationwide DRG implementation in Switzerland, we analyzed time series data of medical patients from 2009 through 2015. We explored whether SwissDRG implementation was associated with an increase in adverse patient outcomes (all-cause 30-day readmission rates and in-hospital mortality) and whether implementation was associated with a trend in LOS observed in the historical control period.

## Methods

The institutional review board of Northwestern Switzerland approved this study and waived informed patient consent owing to the use of deidentified data. This study followed the Strengthening the Reporting of Observational Studies in Epidemiology (STROBE) reporting guideline.^20^

### Participants, Data Sources, and Study Variables

**Figure 1.**
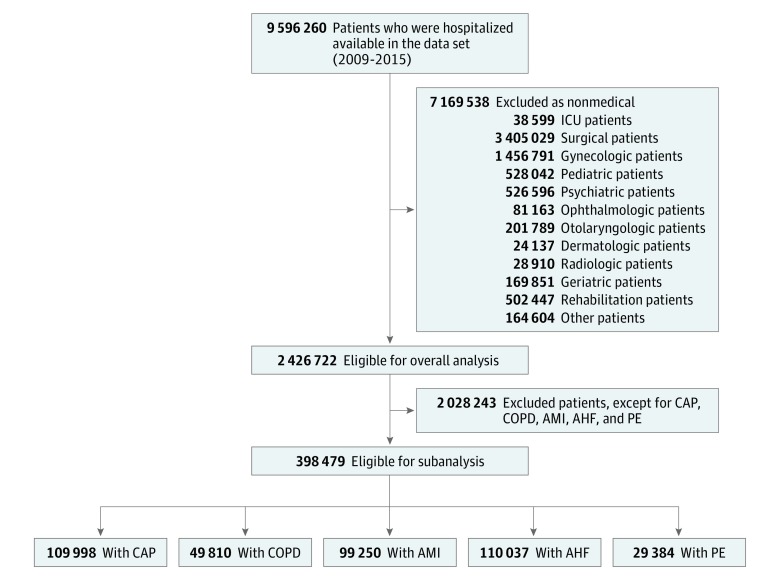
Flow Chart of Study Patients AHF indicates acute heart failure; AMI, acute myocardial infarction; CAP, community-acquired pneumonia; COPD, chronic obstructive pulmonary disease; ICU, intensive care unit; PE, pulmonary embolism.

We included all hospitalizations for adult medical inpatients into the main analysis. For the subanalyses, we included medical hospitalizations for patients presenting with 1 of the following 5 common medical diagnoses,^21^ representing an important inpatient population (Figure 1): community-acquired pneumonia (CAP), exacerbation of chronic obstructive pulmonary disease (COPD), acute myocardial infarction (AMI), acute heart failure (AHF), and pulmonary embolism (PE). We used administrative data (Medizinstatistik) provided by the Swiss Federal Statistical Office between January 1, 2009, and December 31, 2015. The database includes all Swiss inpatient discharge records from acute care, general, and specialty hospitals, excluding hospital units of post–acute care institutions, regardless of payer, and thus, creates a near 100% sample of inpatient discharges in Switzerland.

As SwissDRG implementation was a national effort, we considered all areas in Switzerland. To qualify for inclusion in the subanalysis study cohort, hospitalized patients must have had a principal discharge diagnosis with the following *International Classification of Diseases, Tenth Revision, German Modification* code: J10, J11, J12, J13, J14, J15, J16, J17, J18, and respective CAP subcategories; J44 and COPD subcategories; I21, I22, and AMI subcategories; I11.00, I11.01, I13.00, I13.01, I13.20, I13.21, I50, and subcategories, if applicable to AHF; or I26 and I26.9 (PE). According to the SwissDRG definition, an index admission was an admission in which we evaluated the 18 days after discharge for a readmission into the index hospital. All admissions after 18 days from discharge or admissions into another hospital were evaluated as another index hospitalization.^22^ Information on status of readmission was available for each hospitalization. A single patient may have more than 1 index admission in the study year. Thus, any admission was either an index admission or a readmission, but not both. Length of hospital stay was based on SwissDRG definition, calculated by day of admission and each subsequent day without the day of discharge.^22^

Nonmedical, psychiatric, and nonadult (<18 years of age) patients were excluded from the final analysis. Admissions with transfer into another hospital were considered as a single episode of care. All-cause 30-day readmissions were always attributed to the discharging hospital.

### Exposure

The exposure of interest was the time interval of the nationwide implementation of SwissDRG. The study period was divided into 2 segments: before the implementation of SwissDRG (January 1, 2009, to December 31, 2011) and after the implementation of SwissDRG (January 1, 2012, to December 31, 2015).

### Outcomes

The main quality outcomes were all-cause in-hospital mortality and 30-day readmission rates. The main efficacy outcome was LOS, defined as days spent in the hospital during the index hospitalization (the index admission included all readmissions into the index hospital within 18 days after discharge from the index hospital).

### Statistical Analysis

In this study, we hypothesized that LOS would show a substantially greater decrease after the SwissDRG implementation compared with before the SwissDRG implementation. For this purpose, we compared slopes of monthly time trends before and after the implementation.

We first examined patient and hospital characteristics of the index hospitalization stratified by period of SwissDRG implementation (Table 1). In these descriptive statistics, the index hospitalization was the unit of analysis. We then analyzed the trends in quality outcomes (in-hospital mortality and 30-day readmission rates) and efficacy outcome (LOS) from January 1, 2009, through December 31, 2015. We used an interrupted time series model to examine the linear trends in in-hospital mortality, 30-day readmission rates, and LOS, adjusted for predefined prognostic baseline factors. We included age, sex, hospital teaching level, the Charlson Comorbidity Index, and months of admission as additional covariates for all models. All analyses were adjusted for serial autocorrelation using a Prais-Winsten estimation and the Durbin-Watson statistic.^23^

**Table 1.** Characteristics of Hospitalized Medical Patients

Variable	Before SwissDRG Implementation, 2009-2011, No. (%)	After SwissDRG Implementation, 2012-2015, No. (%)
Sociodemographic		
Hospitalization, No.	1 018 404	1 408 318
Age, median (IQR)	69 (55-80)	70 (56-81)
Male sex	531 226 (52.2)	730 228 (51.9)
Swiss resident	850 773 (83.5)	1 164 185 (82.7)
Hospital teaching level		
Tertiary care hospital	594 814 (58.4)	995 570 (70.7)
Secondary care hospital	387 733 (38.1)	382 833 (27.2)
Other	35 857 (3.5)	29 915 (2.1)
Class of insurance		
General	763 641 (75.0)	1 068 529 (75.9)
Semiprivate	161 150 (15.8)	216 508 (15.4)
Private	93 587 (9.2)	122 370 (8.7)
Unknown	26 (0.0)	911 (0.1)
Morbidity		
Main diagnosis		
Community-acquired pneumonia	43 874 (4.3)	66 124 (4.7)
Exacerbation of COPD	19 046 (1.9)	30 764 (2.2)
Acute myocardial infarction	41 045 (4.0)	58 205 (4.1)
Acute heart failure	44 230 (4.3)	65 807 (4.7)
Pulmonary embolism	12 339 (1.2)	17 045 (1.2)
Charlson Comorbidity Index, mean (SD)	1.4 (2.2)	1.6 (2.3)
Living situation		
Before admission		
At home	888 147 (87.2)	1 227 177 (87.1)
After discharge		
At home	735 832 (73.2)	982 416 (69.8)
Patient outcomes		
Length of hospital stay, mean (SD), d	7.8 (12.8)	7.4 (12.0)
In-hospital mortality	48 780 (4.8)	66 904 (4.8)
30-d readmission	143 190 (14.1)	209 026 (14.8)

Abbreviations: COPD, chronic obstructive pulmonary disease; IQR, interquartile range; SwissDRG, Swiss diagnosis-related groups.

Significance was based on 95% CIs. Statistical analyses were performed from March 1, 2018, to June 30, 2018, and from November 1, 2018, to December 18, 2018, using Stata, version 15.1 (StataCorp LLC).

## Results

### Index Hospitalizations

From January 1, 2009, through December 31, 2015, we identified a total of 2 426 722 medical patients who met our inclusion criteria. Of this total, 1 018 404 patients (41.9%; 531 226 [52.2%] male, median [interquartile range (IQR)] age of 69 [55-80] years) were included in the before-SwissDRG period, and 1 408 318 patients (58.0%; 730 228 [51.9%] male, median [IQR] age of 70 [56-81] years) were included in the after-SwissDRG period. Table 1 shows baseline characteristics stratified by before and after SwissDRG implementation. The median (IQR) age of the overall population was 70 (56-80) years; 52.0% were male and 83.0% were Swiss residents. Two-thirds of patients (1 590 384 [65.5%]) were treated in a tertiary care hospital and 1 832 170 patients (75.5%) had no complementary insurance.

During the overall period, the mean (SD) monthly LOS was 7.6 (12.3) days, with a mean (SD) in-hospital mortality rate of 4.8% (21.3%) and a mean (SD) 30-day readmission rate of 14.5% (35.2%). The number of hospitalizations gradually increased from 333 004 in 2009 to 364 153 in 2015 and involved 222 hospitals. Baseline characteristics stratified for the 5 main diagnoses of interest (CAP, COPD exacerbation, AMI, AHF, and PE) are presented in the eTable in the Supplement.

### Length of Hospital Stay

We found a gradual reduction in mean LOS from unadjusted mean (SD) 8.0 (12.7) days in 2009 to 7.2 (17.3) days in 2015. In the period before SwissDRG implementation, overall LOS decreased at a risk-adjusted slope of –0.0166 days per month (95% CI, –0.0227 to –0.0106 days per month). This decrease in LOS was unchanged after SwissDRG implementation (risk-adjusted slope, –0.0166 days; 95% CI, –0.0223 to –0.0110 days) with a difference between slopes of 0.0000 (95% CI, –0.0072 to 0.0072). Table 2 shows LOS and differences in slopes among the different years adjusted for serial autocorrelation.

**Table 2.** Interrupted Time Series for Risk-Adjusted Length of Hospital Stays, In-Hospital Mortality, and 30-Day Readmission Rates

Clinical Outcome	Years Before SwissDRG Implementation			Years After SwissDRG Implementation				Slope, Monthly Change (95% CI), d^a^		Differences Between Slopes (95% CI)
	2009	2010	2011	2012	2013	2014	2015	2009-2011	2012-2015	
**Overall**										
Length of hospital stay, mean (SD), d	8.0 (12.7)	7.8 (11.4)	7.7 (14.1)	7.6 (10.3)	7.4 (9.2)	7.3 (8.7)	7.2 (17.3)	−0.0166 (−0.0227 to −0.0106)	−0.0166 (−0.0223 to −0.0110)	0.0000 (−0.0072 to 0.0072)
In-hospital mortality, %	4.9	4.8	4.7	4.9	4.8	4.7	4.6	−0.021 (−0.0283 to −0.0141)	−0.0327 (−0.0390 to −0.0264)	−0.0115 (−0.0190 to −0.0039)
30-d readmission, %	14.4	14.1	13.8	14.3	15.0	15.0	15.0	−0.0303 (−0.0389 to −0.0216)	0.0036 (−0.0049 to 0.0121)	0.0339 (0.0254 to 0.0423)
**Community-acquired Pneumonia**										
Length of hospital stay, mean (SD), d	9.2 (8.9)	9.1 (7.3)	9.0 (7.1)	9.0 (7.2)	8.7 (6.6)	8.4 (6.3)	8.3 (6.5)	−0.0102 (−0.0205 to 0.0001)	−0.0193 (−0.0286 to −0.0099)	−0.0091 (−0.0219 to 0.0038)
In-hospital mortality, %	6.2	5.4	5.8	5.7	5.3	4.8	4.9	−0.0130 (−0.0408 to 0.0148)	−0.0230 (−0.0418 to −0.0042)	−0.0100 (−0.0406 to 0.0206)
30-d readmission, %	10.3	11.1	10.5	11.0	11.4	11.3	11.5	−0.0069 (−0.0459 to 0.0320)	0.0035 (−0.0330 to 0.0400)	0.0104 (−0.0365 to 0.0574)
**COPD Exacerbation**										
Length of hospital stay, mean (SD), d	10.2 (8.0)	9.6 (8.5)	9.4 (7.1)	9.4 (7.7)	8.8 (7.0)	8.9 (7.0)	8.5 (6.8)	−0.0310 (−0.0440 to −0.0181)	−0.0246 (−0.0371 to −0.0122)	0.0064 (−0.0104 to 0.0232)
In-hospital mortality, %	3.9	3.3	3.5	4.2	4.3	4.3	4.0	−0.0300 (−0.0534 to −0.0066)	−0.0393 (−0.0601 to −0.0184)	−0.0093 (−0.0341 to 0.0155)
30-d readmission, %	17.8	16.5	16.9	17.1	19.1	17.3	19.3	0.0044 (−0.0856 to 0.0943)	0.0264 (−0.0663 to 0.1190)	0.0220 (−0.0982 to 0.1423)
**Acute Myocardial Infarction**										
Length of hospital stay, mean (SD), d	6.3 (7.1)	5.9 (6.7)	5.8 (6.4)	6.0 (6.3)	5.5 (5.9)	5.5 (6.0)	5.2 (5.4)	−0.0197 (−0.0278 to −0.0115)	−0.0192 (−0.0245 to −0.0140)	0.0004 (−0.0081 to 0.0090)
In-hospital mortality, %	6.0	5.2	5.1	5.5	5.3	4.7	4.5	−0.0397 (−0.0565 to −0.0229)	−0.0363 (−0.0525 to −0.0201)	0.0034 (−0.0172 to 0.0239)
30-d readmission, %	14.2	14.1	12.8	20.5	22.6	22.6	23.2	−0.0736 (−0.1230 to −0.0242)	0.0408 (0.0052 to 0.0764)	0.1144 (0.0617 to 0.1671)
**Acute Heart Failure**										
Length of hospital stay, mean (SD), d	10.1 (8.2)	10.0 (8.2)	9.8 (7.7)	9.7 (7.6)	9.7 (7.9)	9.7 (7.7)	9.5 (7.7)	−0.0218 (−0.0309 to −0.0127)	−0.0178 (−0.0261 to −0.0094)	0.0040 (−0.0054 to 0.0134)
In-hospital mortality, %	7.6	7.8	7.7	7.7	7.7	7.7	7.7	−0.0241 (−0.0736 to 0.0254)	−0.0079 (−0.0527 to 0.0370)	0.0162 (−0.0356 to 0.0680)
30-d readmission, %	14.4	13.9	14.0	15.0	16.4	16.8	16.3	−0.0513 (−0.0936 to −0.0090)	−0.0387 (−0.0835 to 0.0061)	0.0126 (−0.0358 to 0.0610)
**Pulmonary Embolism**										
Length of hospital stay, mean (SD), d	8.1 (6.5)	8.0 (6.3)	7.6 (6.3)	7.5 (5.8)	7.2 (6.3)	7.0 (6.1)	6.6 (5.7)	−0.0217 (−0.0339 to −0.0094)	−0.0274 (−0.0366 to −0.0183)	−0.0057 (−0.0196 to 0.0082)
In-hospital mortality, %	4.5	4.9	4.4	4.0	4.1	4.3	4.0	−0.0462 (−0.0855 to −0.0068)	−0.0307 (−0.0632 to 0.0018)	0.0155 (−0.0323 to 0.0633)
30-d readmission, %	8.1	7.8	7.5	8.3	9.1	9.3	9.2	−0.0528 (−0.1140 to 0.0084)	−0.0027 (−0.0553 to 0.0498)	0.0501 (−0.0144 to 0.1146)

Abbreviations: COPD, chronic obstructive pulmonary disease; SwissDRG, Swiss diagnosis-related group.

^a^ Slopes represent the slope of the regression line in the corresponding period, representing the mean change in length of hospital stay, in-hospital mortality, and 30-day readmission rates over a month in this period.

**Figure 2.**
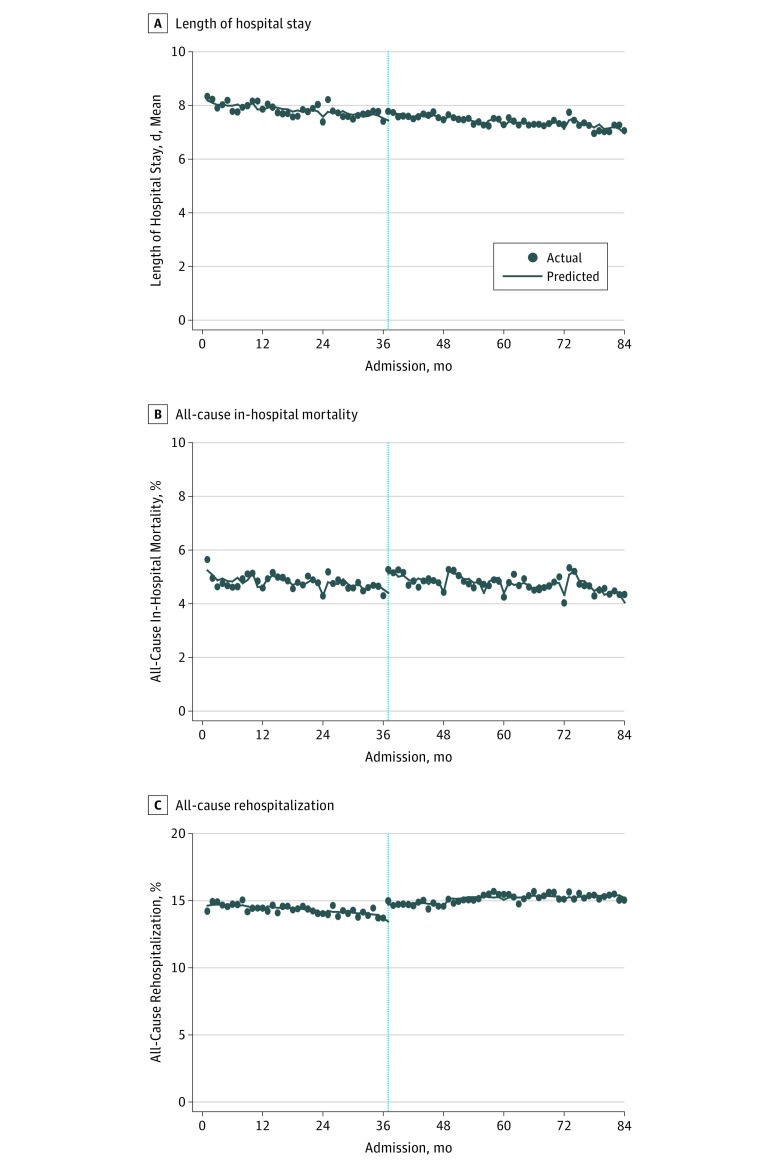
Time Trends in Risk-Adjusted Length of Hospital Stay, In-Hospital Mortality, and 30-Day Readmission Rates A, Linear trends in mean monthly risk-adjusted length of hospital stay. B, Linear trends in all-cause in-hospital mortality. C, Linear trends in all-cause 30-day readmission rates. The vertical dashed line denotes January 1, 2012, the date of SwissDRG reimbursement implementation. The trend line is fitted on the basis of predictions of truncated time series models for the 2 periods (before implementation: January 1, 2009, to December 31, 2011; after implementation: January 1, 2012, to December 31, 2015). The interrupted time series model is tested for serial autocorrelation; all analyses were adjusted for serial autocorrelation using a Prais-Winsten estimation and the Durbin-Watson statistic. Risk adjustment was made for patient age, sex, Charlson Comorbidity Index, teaching level of hospital, and month of hospital admission.

In subgroup analyses involving patients with CAP, COPD exacerbation, AMI, AHF, or PE, SwissDRG implementation was associated with a statistically significant reduction per month in LOS (CAP: slope, −0.0193 days [95% CI, −0.0286 to −0.0099 days]; COPD exacerbation: slope, −0.0246 days [95% CI, −0.0371 to −0.0122 days]; AMI: slope, −0.0192 days [−95% CI, 0.0245 to −0.0140 days]; AHF: slope, −0.0178 days [95% CI, −0.0261 to −0.0094 days]; PE: slope, −0.0274 days [95% CI, −0.0366 to −0.0183 days]); however, LOS was not different in the period before SwissDRG implementation. All time series analyses of risk-adjusted LOS are illustrated in Figure 2 and eFigures 1-5 in the Supplement.

### In-Hospital Mortality and 30-Day Readmission

The overall risk-adjusted all-cause in-hospital mortality declined from 4.9% in 2009 to 4.6% in 2015. At the same time, risk-adjusted 30-day readmission rate increased from 14.4% in 2009 to 15.0% in 2015, with an intercept of approximately 1% between 2011 and 2012. The time series analyses of all-cause in-hospital mortality and 30-day readmissions in the 2 periods are shown in Table 2 and Figure 2. We found a larger reduction in overall risk-adjusted in-hospital mortality after SwissDRG implementation (difference between monthly slopes before and after implementation, –0.0115%; 95% CI, –0.0190% to –0.0039%); however, a statistically significant increase in risk-adjusted 30-day readmissions was found in the period after SwissDRG implementation compared with the period before SwissDRG implementation (change in monthly slope, 0.0339%; 95% CI, 0.0254%-0.0423%). We observed a gradual reduction in risk-adjusted in-hospital mortality after SwissDRG implementation in patients with CAP, COPD exacerbation, and AMI, but no difference was observed in the control period before implementation. In-hospital mortality did not change in patients with AHF (7.7%) and PE (4.0%) after 2011.

The increase in 30-day readmission after SwissDRG implementation was strongest for patients with AMI (change in monthly slope, 0.0408%; 95% CI, 0.0052%-0.0764%), with a difference between slopes of 0.1144% (95% CI, 0.0617%-0.1671%). In addition, we found a large intercept of approximately 8% between 2011 and 2012 in patients with a previous AMI. However, risk-adjusted 30-day readmission rates for patients with CAP, COPD exacerbation, AHF, and PE did not change between 2012 and 2015. The rates also did not change when compared with the historical control period between 2009 and 2011 (eFigures 1-5 in the Supplement).

## Discussion

The findings of this large study that used administrative data of adult patients hospitalized for a medical condition in Switzerland are 2-fold. First, the change in hospital reimbursement by implementation of SwissDRG in 2012 was associated with a significant increase in hospital readmission rates and, at the same time, lower in-hospital mortality. Second, although we found a gradual reduction in LOS from 8.0 days in 2009 to 7.2 days in 2015, the introduction of SwissDRG did not appear to be associated with this reduction with similar monthly reductions in LOS in the periods before and after change in hospital reimbursement.

The SwissDRG system was introduced in 2012 to improve the efficacy of in-hospital treatment by providing financial incentives for reducing LOS, as payment remains similar but use of in-hospital resources decreases with shorter LOS. We hypothesized that the introduction of SwissDRG would make the trend of shortening LOS substantially greater. The main goal of this study was to investigate the associations of SwissDRG with health-related outcomes, particularly in-hospital mortality and 30-day readmission rates. Although the gradual decrease in LOS was not apparently affected by the implementation of SwissDRG in 2012, it remains unclear whether this gradual decrease in LOS would have been observed if the initial fee-for-service system were kept. In addition, we found no evidence that the gradual reduction in LOS was associated with a potential increase in in-hospital mortality, but 30-day readmission rates rose after SwissDRG implementation. Interpretation of these safety data are challenging as we do not know the natural history if the reimbursement system were not changed.

Because our analysis relied on administrative data, we were not able to investigate the association with 30-day mortality or other patient-centered outcomes.^18,24,25,26,27^ The validity of in-hospital mortality as a quality measure has been questioned, as physicians may decide to discharge patients with terminal illness to palliative care centers. A shorter LOS per se may explain the reduced in-hospital mortality owing to less time at risk. Yet, 30-day mortality was not available in our data set to enable us to test these hypotheses. As we found higher rates of hospital 30-day readmission, including short stays of fewer than 24 hours, our findings do not refute the hypothesis that the gradual reduction in LOS negatively affects patient outcome.

Similarly, it is still unclear if the SwissDRG implementation is associated with cost savings, as LOS was apparently not affected by SwissDRG implementation, but readmissions significantly increased since 2012. As we do not have postdischarge mortality data, the interpretation of readmission rates is also challenging. Further evaluation of other patient outcome measures, such as postdischarge mortality, emergency department visits without formal readmission, patient reported experience measures, and patient reported outcome measures, would provide evidence and improve confidence in our analyses.

To our knowledge, this study is the first large-scale study to use administrative data to investigate the association of SwissDRG reimbursement with patient outcomes. Outside of the DRG reimbursement system, similar strategies were followed by other countries, including the United States with the implementation of the Affordable Care Act and related reforms. In a study from the United States involving patients with AMI and AHF,^28,29^ higher hospital-level 30-day-episode payments were associated with lower patient mortality. This finding had important implications for policies that incentivized reduction in payments without considering value.^28,29^ Whether patient outcomes will be compromised in Switzerland if incentives are reduced by the implementation of the SwissDRG remains to be seen. Readmission rates are regarded as important quality measures, as in some cases they may be a result of premature discharge or adverse events. Penalties in the Hospital Readmissions Reduction Program as part of the Affordable Care Act in the United States were based on the readmission rates to improve efficiency and quality of care.^30^ In this study, we found an increase in readmission rates over the years, which is inconsistent with findings in previous smaller studies.^14,16,17,25,31^ Nonetheless, as there are inherent differences within the Swiss health care systems, the association of the SwissDRG implementation with LOS, in-hospital mortality, and 30-day readmission rates remains speculative. Previous studies were strongly limited by selection bias and small sample sizes, and they revealed heterogeneous results.^24,31,32^

### Limitations

This study has limitations. Investigating the associations of a national change in hospital reimbursement with clinical outcomes is challenging and prone to confounding. The nonexperimental design of the study cannot draw a firm causal link between SwissDRG implementation and outcomes of interest. Nonetheless, we used an interrupted time series design that tests for serial autocorrelations, which allowed us to study time trends in an adjusted analysis for important prognostic factors and to examine the associations between SwissDRG implementation and outcomes. This approach is in line with that used in previous studies from other countries.^16,17,19,24,25,31,32^ Still, whether these results are only explained by the introduction of SwissDRG or whether other factors, including medical progress or patient selection, affected the results remains unclear.^9,16,24^ Decrease in LOS could also be underestimated, as the lower baseline LOS in December 2011 compared with January 2009 made it more challenging to reduce LOS after SwissDRG implementation. In addition, the association of SwissDRG implementation with patient outcomes may have been underestimated, as some hospitals already used a DRG-type reimbursement system before SwissDRG was introduced nationwide in 2012. Unfortunately, we did not have information on the SwissDRG dissemination before the official start on January 1, 2012. Nonetheless, our results suggest that the introduction of SwissDRG may have stimulated behavioral changes in some hospitals, which led to more efficient discharge planning.

Furthermore, the attribution of changes in patient outcomes to a specific time point is also confounded in any statistical analysis because of possible time lags between SwissDRG implementation and any resulting change in the patient outcome. In line with this aspect, we found a step increase of 30-day readmissions after SwissDRG implementation, especially in hospitalizations for AMI. The reasons for this finding might be an increase in performing a second coronary angiography in cases of multivessel disease as well as a shorter LOS and a certain imprecision in coding diagnoses before 2012. Last, we did not have any information on the outpatient setting, and we were not able to draw any conclusions about intermediate or long-term mortality rates.

Because a quality assurance system, the necessary natural partner of DRG, was not strengthened or closely attached by Swiss law and by the delegated corporate bodies, the potential risks of the SwissDRG introduction are officially monitored on a macro level only and not on a differentiated clinical level. Clinicians’ self-responsibility to monitor the quality of care and to allocate resources for their processes is inevitable on a micro level and as outlined by this study.

## Conclusions

In adult patients hospitalized for a medical condition in Switzerland, SwissDRG implementation appears to be associated with an increase in readmission rates and lower in-hospital mortality but was not associated with the decrease in length of hospital stay observed in the historical control period. More research is needed to understand the association of hospital reimbursement with in-hospital care to improve both as well as the effectiveness and quality of the health care system.

## Notes

Supplement. **eTable.** Characteristics of Patients with Community-acquired Pneumonia, COPD, Acute Myocardial Infarction, Acute Heart Failure, Pulmonary Embolism **eFigure 1. **Time Trends in Risk-adjusted Length of Hospital Stay, Mortality, and Readmission in Community-acquired Pneumonia Patients **eFigure 2.** Time Trends in Risk-adjusted Length of Hospital Stay, Mortality, and Readmission in exacerbated COPD Patients **eFigure 3.** Time Trends in Risk-adjusted Length of Hospital Stay, Mortality, and Readmission in Acute Myocardial Infarction Patients **eFigure 4.** Time Trends in Risk-adjusted Length of Hospital Stay, Mortality, and Readmission in Acute Heart Failure Patients **eFigure 5.** Time Trends in Risk-adjusted Length of Hospital Stay, Mortality, and Readmission in Pulmonary Embolism Patients
