# Supplementary material for: Association of the Swiss Diagnosis-Related Group Reimbursement System With Length of Stay, Mortality, and Readmission Rates in Hospitalized Adult Patients
Source: JAMA Netw Open. 2019 Feb 15;2(2):e188332. doi: 10.1001/jamanetworkopen.2018.8332 (PMC6484617; doi:10.1001/jamanetworkopen.2018.8332)
Supplement: Supplement. — eTable. Characteristics of Patients with Community-acquired Pneumonia, COPD, Acute Myocardial Infarction, Acute Heart Failure, Pulmonary Embolism eFigure 1. Time Trends in Risk-adjusted Length of Hospital Stay, Mortality, and Readmission in Community-acquired Pneumonia Patients eFigure 2. Time Trends in Risk-adjusted Length of Hospital Stay, Mortality, and Readmission in exacerbated COPD Patients eFigure 3. Time Trends in Risk-adjusted Length of Hospital Stay, Mortality, and Readmission in Acute Myocardial Infarction Patients eFigure 4. Time Trends in Risk-adjusted Length of Hospital Stay, Mortality, and Readmission in Acute Heart Failure Patients eFigure 5. Time Trends in Risk-adjusted Length of Hospital Stay, Mortality, and Readmission in Pulmonary Embolism Patients [file jamanetwopen-2-e188332-s001.pdf]

Supplementary Online Content

Kutz A, Gut L, Ebrahimi F, Wagner U, Schuetz P, Mueller B. Association of the Swiss diagnosis-related group reimbursement system with length of stay, mortality, and readmission rates in hospitalized adult patients. *JAMA Netw Open.* 2019;2(2): e188332. doi:10.1001/jamanetworkopen.2018.8332

eTable. Characteristics of Patients with Community-acquired Pneumonia, COPD exacerbation, Acute Myocardial Infarction, Acute Heart Failure, Pulmonary Embolism

eFigure 1. Time Trends in Risk-adjusted Length of Hospital Stay, Mortality, and Readmission in Community-acquired Pneumonia Patients

eFigure 2. Time Trends in Risk-adjusted Length of Hospital Stay, Mortality, and Readmission in exacerbated COPD Patients

eFigure 3. Time Trends in Risk-adjusted Length of Hospital Stay, Mortality, and Readmission in Acute Myocardial Infarction Patients

eFigure 4. Time Trends in Risk-adjusted Length of Hospital Stay, Mortality, and Readmission in Acute Heart Failure Patients

eFigure 5. Time Trends in Risk-adjusted Length of Hospital Stay, Mortality, and Readmission in Pulmonary Embolism Patients

This supplementary material has been provided by the authors to give readers additional information about their work.

eTable. Characteristics of Patients with Community-acquired Pneumonia, COPD exacerbation, Acute Myocardial Infarction, Acute Heart Failure, Pulmonary Embolism

	**Before SwissDRG Implementation (2009 to 2011)**	**After SwissDRG Implementation (2012 to 2015)**
**Socio-demographics**		
Hospitalizations, n	160`534	237`945
Age, median (IQR)	74 (63, 83)	75 (64, 83)
Male gender, n (%)	92`600 (57.7)	136`057 (57.2)
Swiss residents, n (%)	136`707 (85.2)	199`861 (84.0)
Hospital teaching level, n (%)		
Tertiary care hospital	94`664 (59.0)	169`393 (71.2)
Secondary care hospital	61`331 (38.2)	65`216 (27.4)
Other	4`539 (2.8)	3`336 (1.4)
Class of insurance, n (%)		
general	123`773 (77.1)	185`454 (77.9)
semiprivate	24`145 (15.0)	34`395 (14.5)
private	12616 (7.9)	18`091 (7.6)
unknown	0 (0.0)	5 (0.0)
**Morbidity, n (%)**		
Main diagnosis		
Community-acquired pneumonia	43`874 (27.3)	66`124 (27.8)
Exacerbation of COPD	19`046 (11.9)	30`764 (12.9)
Acute myocardial infarction	41`045 (25.6)	58`205 (24.5)
Acute heart failure	44`230 (27.6)	65`807 (27.7)
Pulmonary embolism	12`339 (7.7)	17`045 (7.2)
Charlson Comorbidity Index, mean (SD)	1.6 (1.6)	1.9 (1.7)
**Living situation, n (%)**		
Before admission		
At home	132`526 (82.6)	197`038 (82.8)
After discharge		
At home	106`529 (66.4)	156`552 (65.8)
**Patient outcomes**		
Length of stay, mean (SD), d	8.5 (7.7)	8.1 (7.0)
In-hospital mortality, n (%)	9`408 (5.9)	13`367 (5.6)
30-day readmission, n (%)	20`725 (12.9)	38`193 (16.1)

COPD, Chronic obstructive pulmonary disease; IQR, Interquartile range; SD, standard deviation.

©2019 Kutz A et al. *JAMA Network Open.*

eFigure 1. Time Trends in Risk-adjusted Length of Hospital Stay, Mortality, and Readmission in Community-acquired Pneumonia Patients

Linear trends in mean monthly risk-adjusted (A) length of hospital stay, (B) all-cause in-hospital mortality, and (C) all-cause 30-day readmission rates. The vertical line denote January 1, 2012 to be proximate to date of implementation of SwissDRG reimbursement. Trend line is fitted based on predictions of truncated time series models for the two periods above. The interrupted time series model is tested for serial autocorrelation. Risk adjustment was made for patient age, sex, Charlson Comorbidity Index, teaching level of center, and month of hospital admission.

eFigure 1A

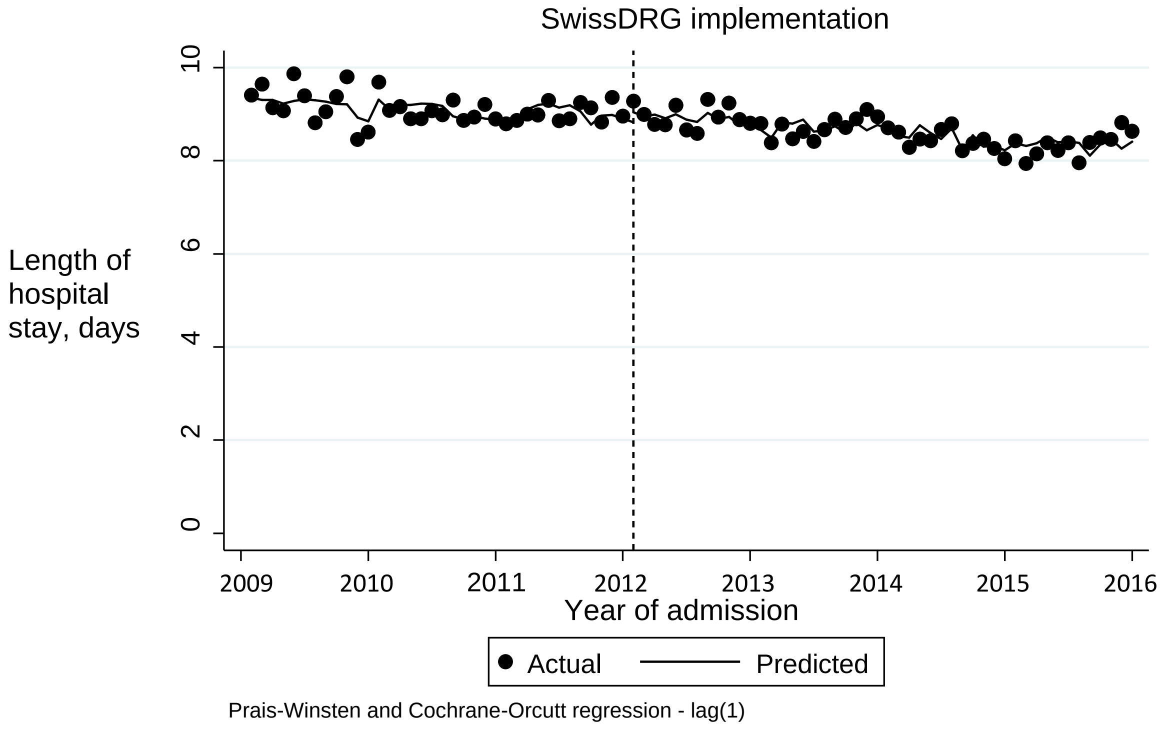

©2019 Kutz A et al. *JAMA Network Open.*

eFigure 1B

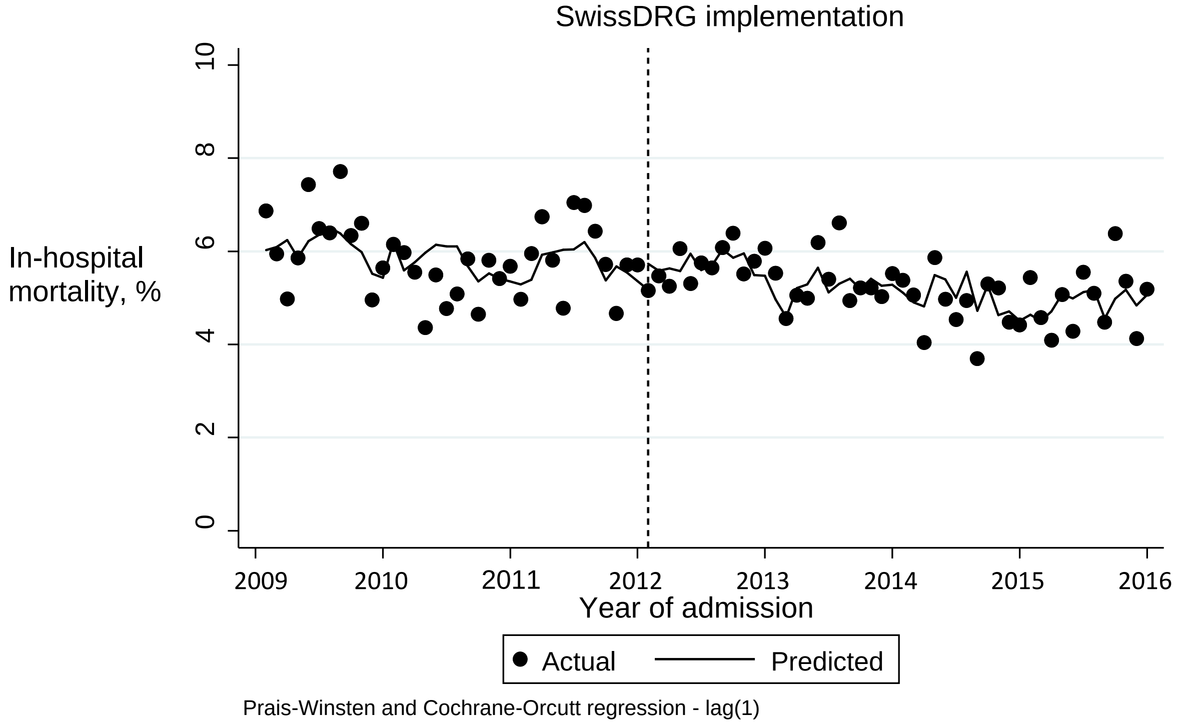

eFigure 1C

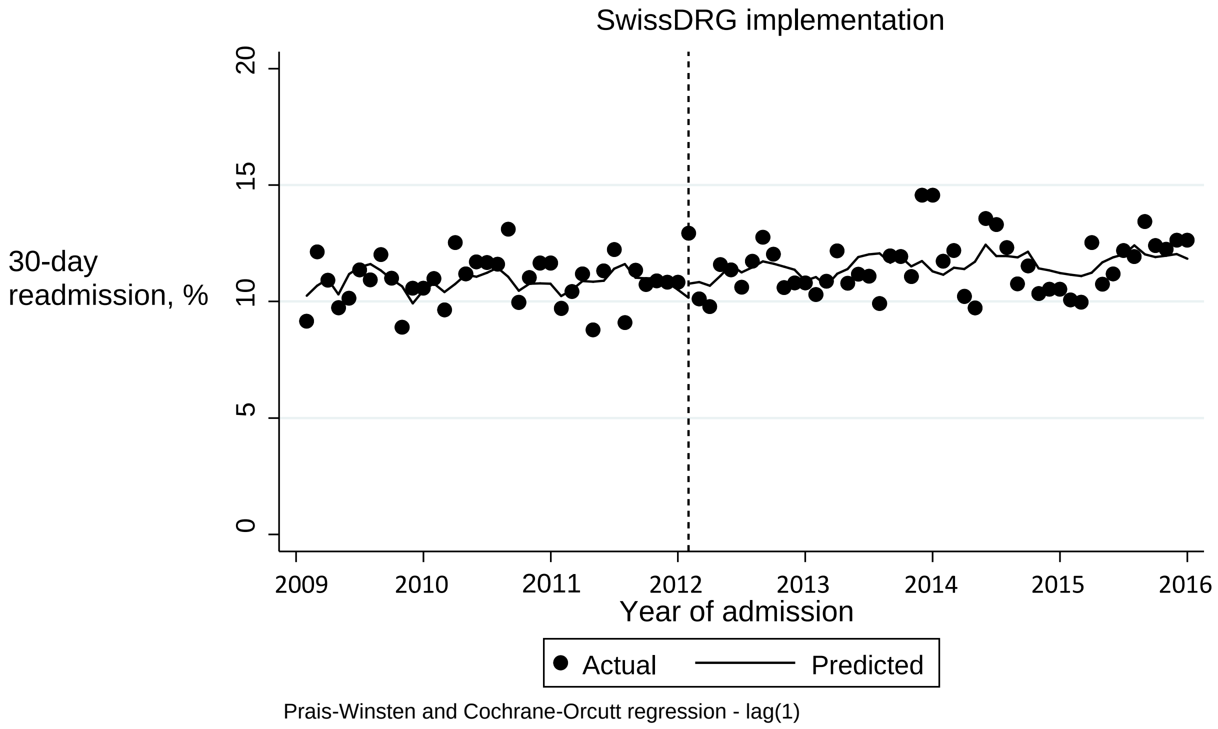

©2019 Kutz A et al. *JAMA Network Open.*

eFigure 2. Time Trends in Risk-adjusted Length of Hospital Stay, Mortality, and Readmission in exacerbated COPD Patients

Linear trends in mean monthly risk-adjusted (A) length of hospital stay, (B) all-cause in-hospital mortality, and (C) all-cause 30-day readmission rates. The vertical line denote January 1, 2012 to be proximate to date of implementation of SwissDRG reimbursement. Trend line is fitted based on predictions of truncated time series models for the two periods above. The interrupted time series model is tested for serial autocorrelation. Risk adjustment was made for patient age, sex, Charlson Comorbidity Index, teaching level of center, and month of hospital admission.

eFigure 2A

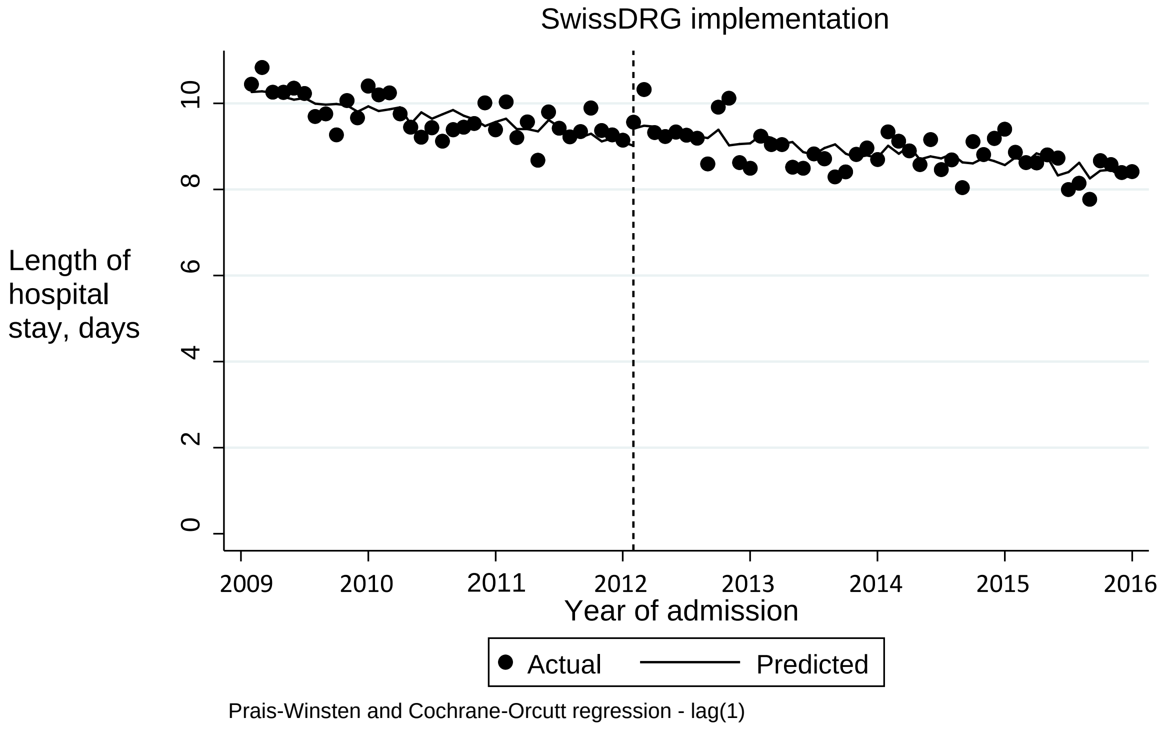

©2019 Kutz A et al. *JAMA Network Open.*

eFigure 2B

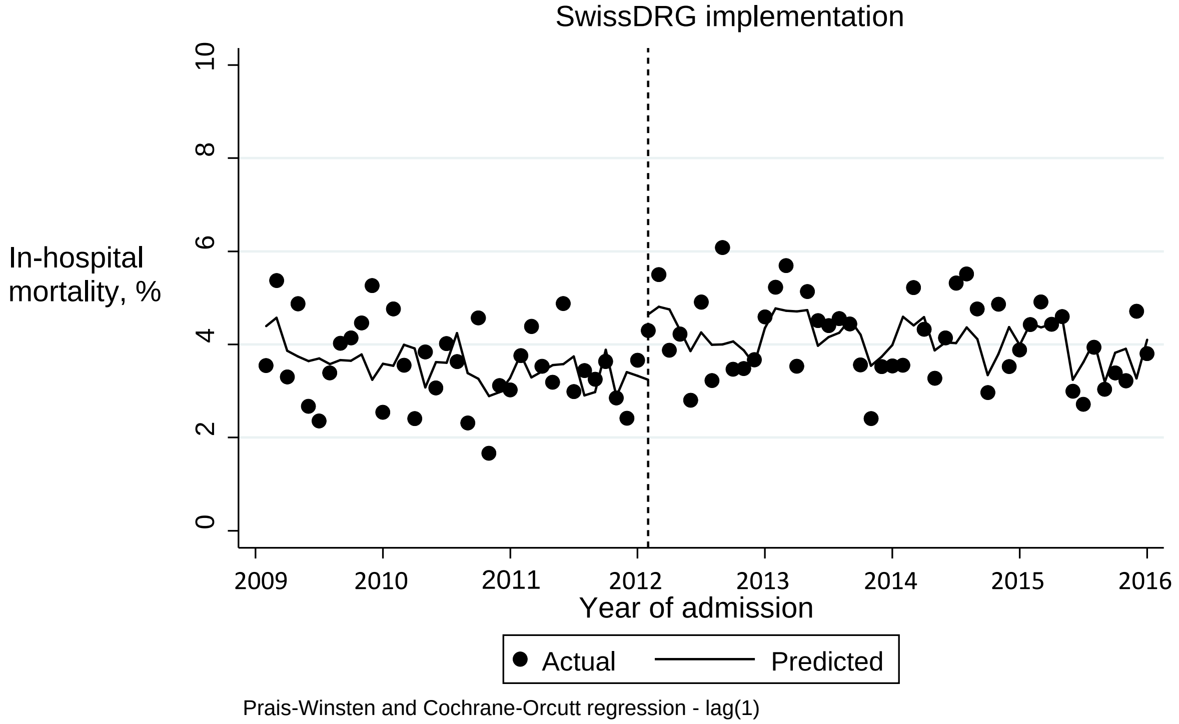

eFigure 2C

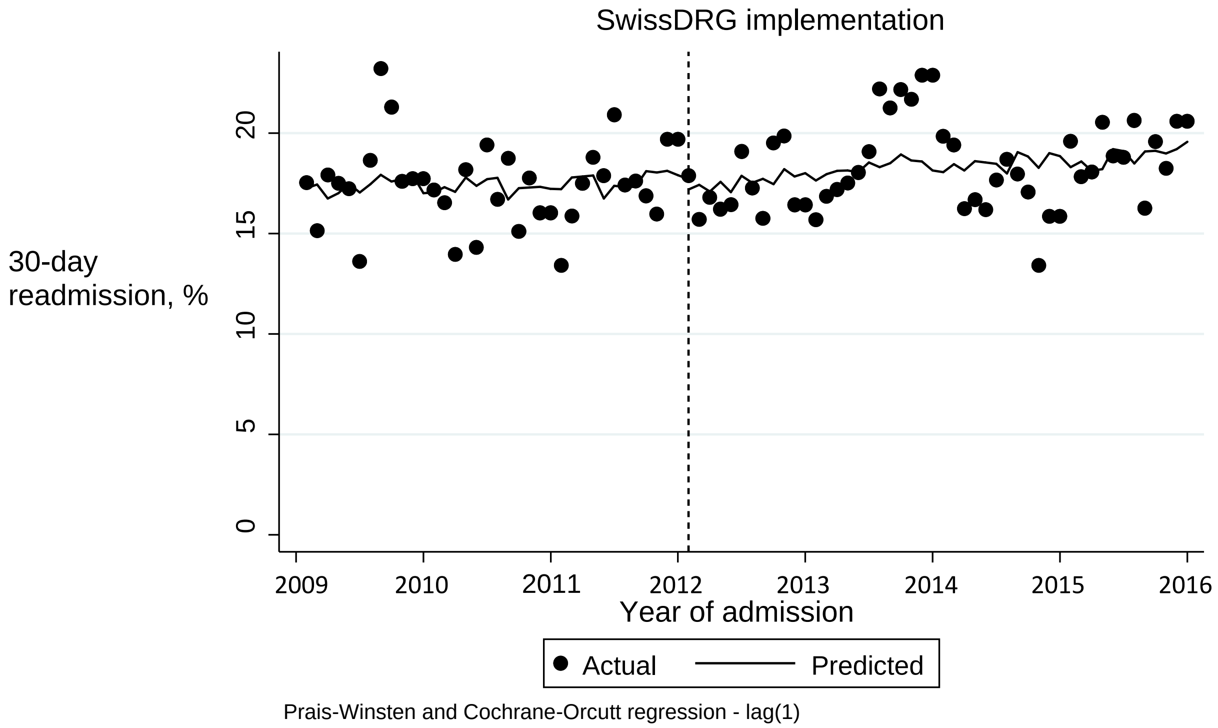

©2019 Kutz A et al. *JAMA Network Open.*

eFigure 3. Time Trends in Risk-adjusted Length of Hospital Stay, Mortality, and Readmission in Acute Myocardial Infarction Patients

Linear trends in mean monthly risk-adjusted (A) length of hospital stay, (B) all-cause in-hospital mortality, and (C) all-cause 30-day readmission rates. The vertical line denote January 1, 2012 to be proximate to date of implementation of SwissDRG reimbursement. Trend line is fitted based on predictions of truncated time series models for the two periods above. The interrupted time series model is tested for serial autocorrelation. Risk adjustment was made for patient age, sex, Charlson Comorbidity Index, teaching level of center, and month of hospital admission.

eFigure 3A

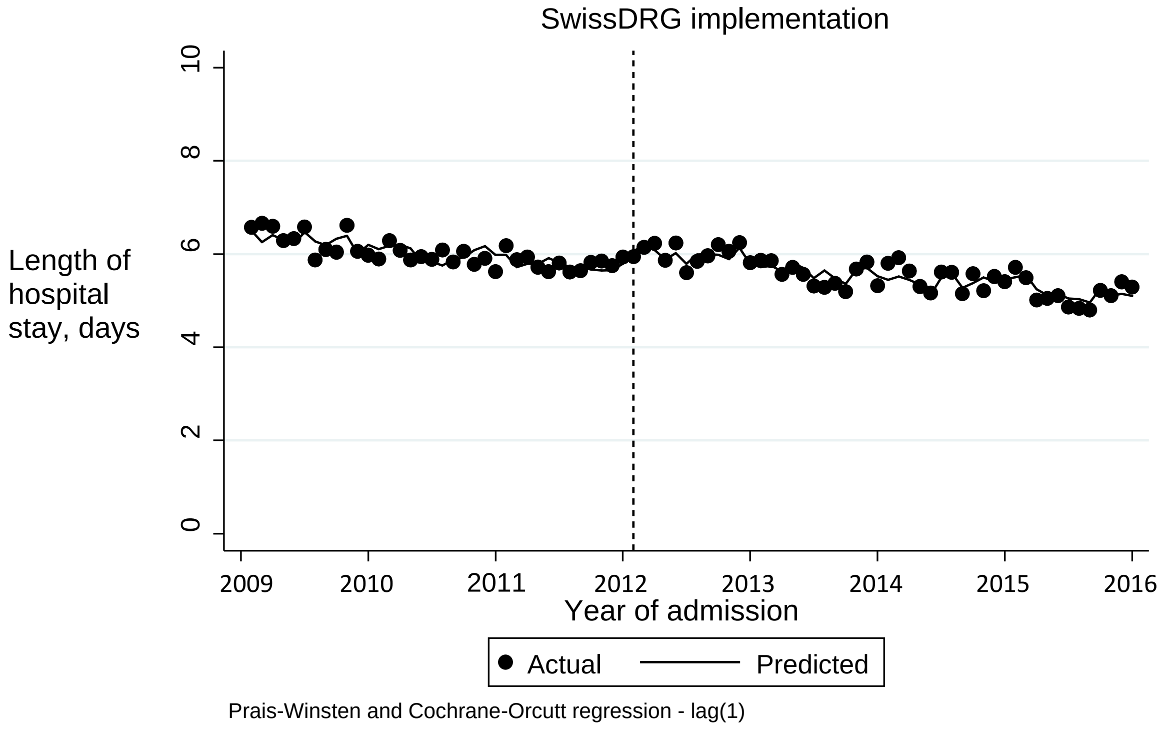

©2019 Kutz A et al. *JAMA Network Open.*

eFigure 3B

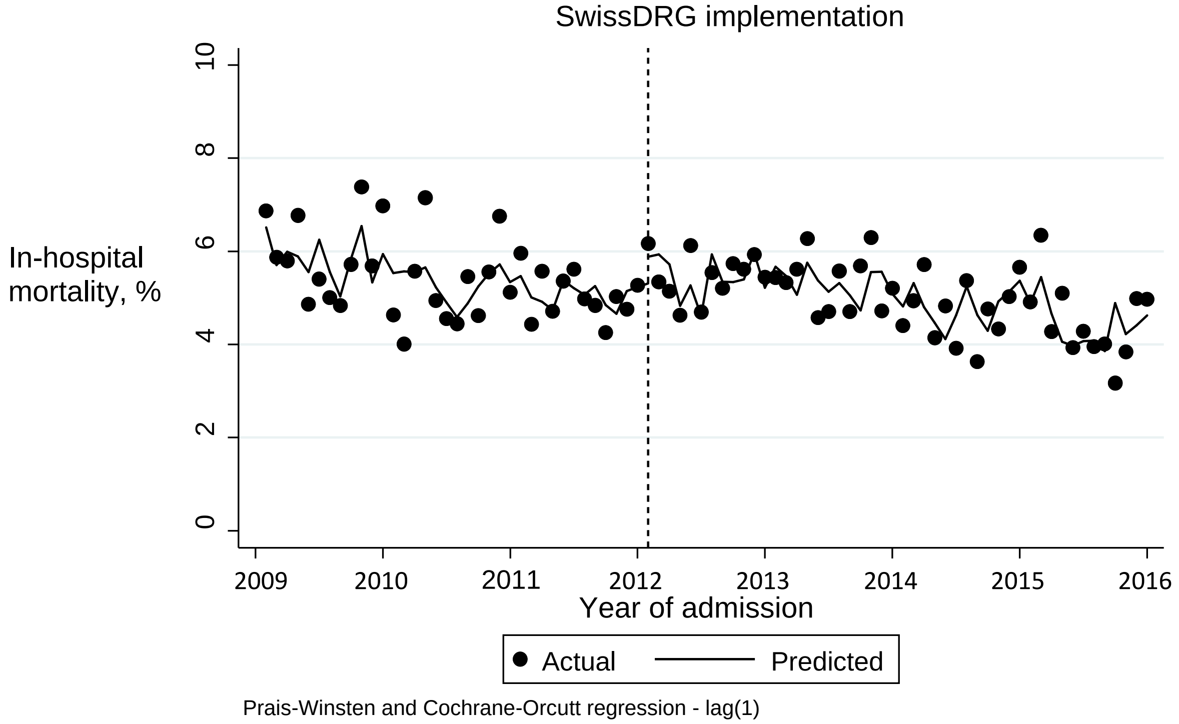

eFigure 3C

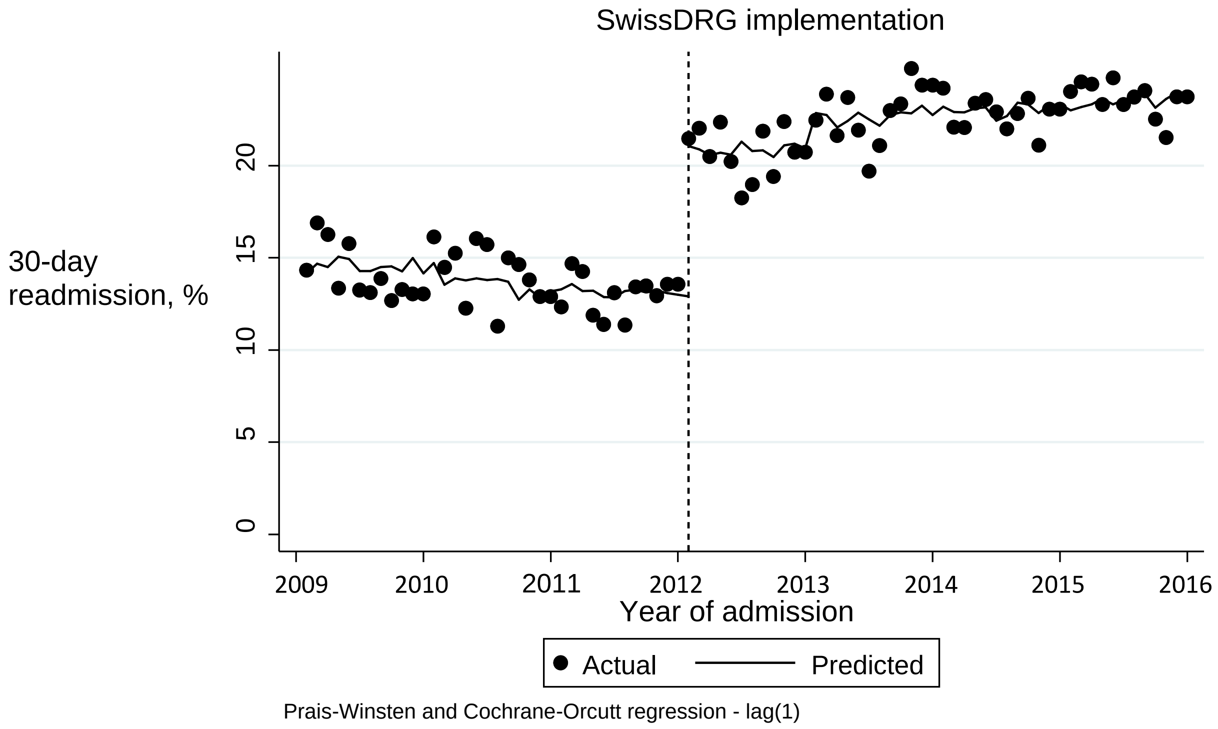

©2019 Kutz A et al. *JAMA Network Open.*

eFigure 4. Time Trends in Risk-adjusted Length of Hospital Stay, Mortality, and Readmission in Acute Heart Failure Patients

Linear trends in mean monthly risk-adjusted (A) length of hospital stay, (B) all-cause in-hospital mortality, and (C) all-cause 30-day readmission rates. The vertical line denote January 1, 2012 to be proximate to date of implementation of SwissDRG reimbursement. Trend line is fitted based on predictions of truncated time series models for the two periods above. The interrupted time series model is tested for serial autocorrelation. Risk adjustment was made for patient age, sex, Charlson Comorbidity Index, teaching level of center, and month of hospital admission.

eFigure 4A

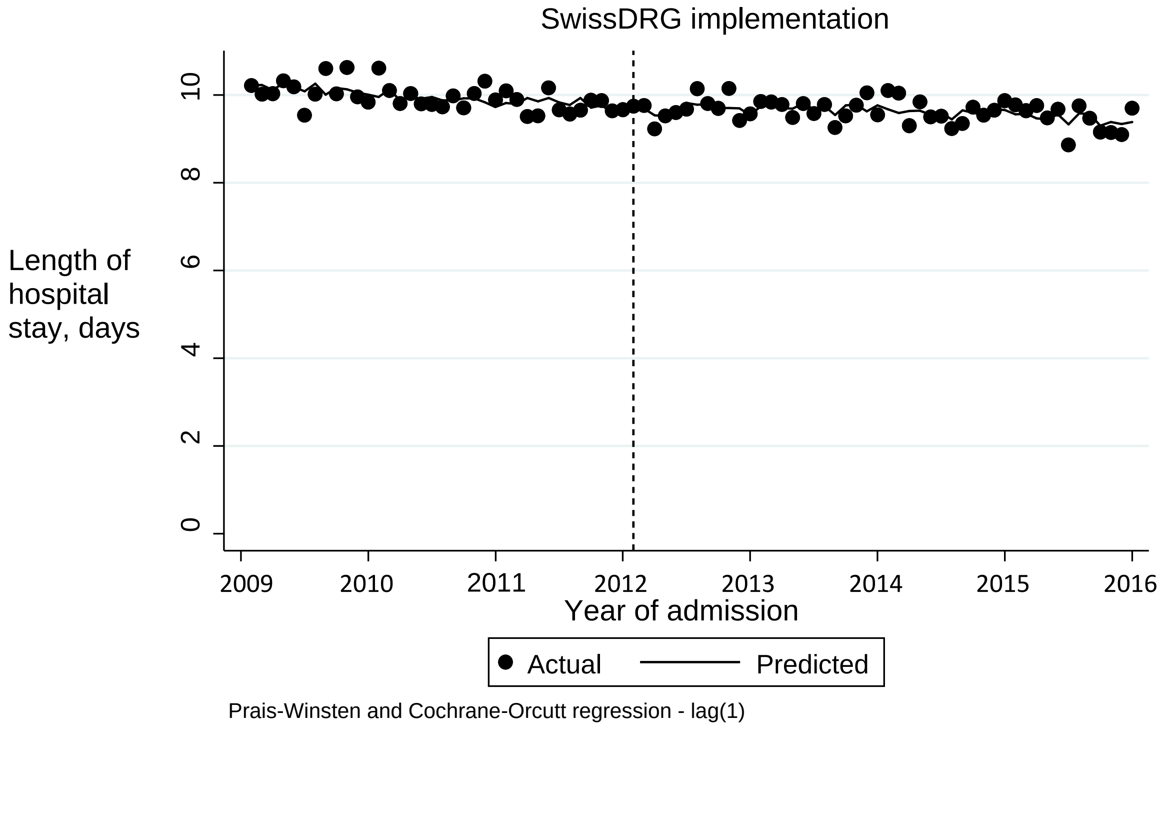

©2019 Kutz A et al. *JAMA Network Open.*

eFigure 4B

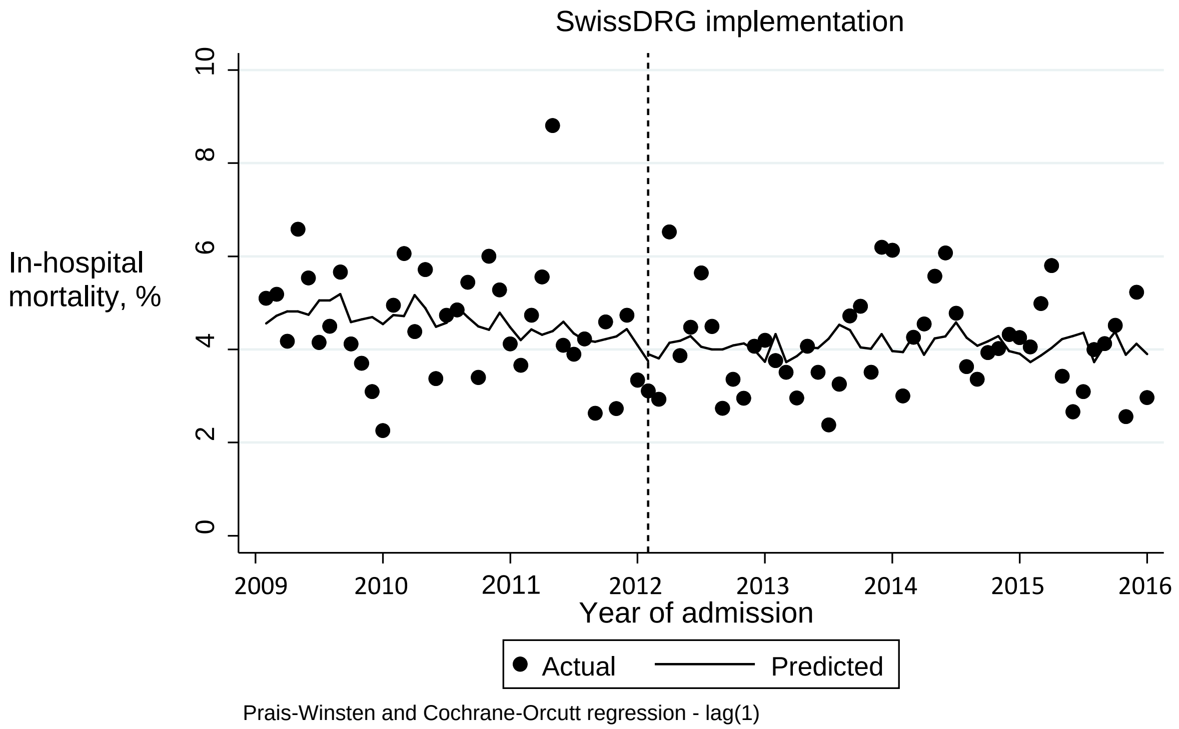

eFigure 4C

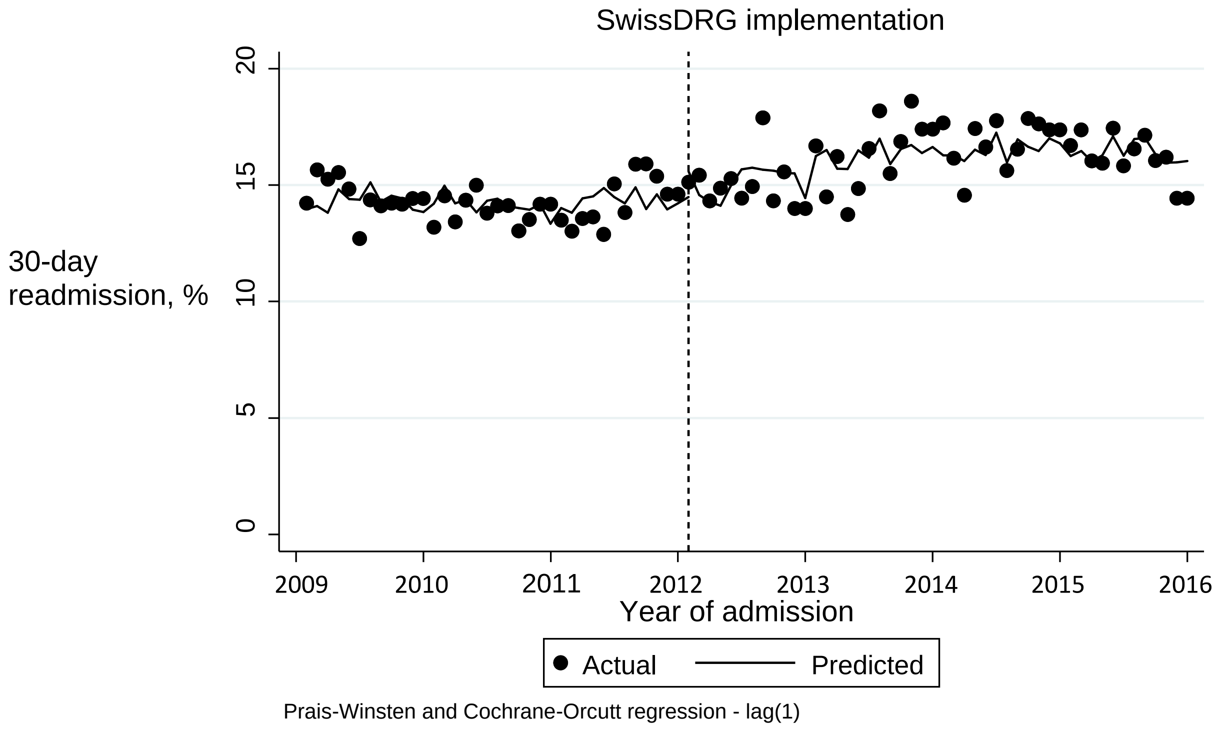

©2019 Kutz A et al. *JAMA Network Open.*

eFigure 5. Time Trends in Risk-adjusted Length of Hospital Stay, Mortality, and Readmission in Pulmonary Embolism Patients

Linear trends in mean monthly risk-adjusted (A) length of hospital stay, (B) all-cause in-hospital mortality, and (C) all-cause 30-day readmission rates. The vertical line denote January 1, 2012 to be proximate to date of implementation of SwissDRG reimbursement. Trend line is fitted based on predictions of truncated time series models for the two periods above. The interrupted time series model is tested for serial autocorrelation. Risk adjustment was made for patient age, sex, Charlson Comorbidity Index, teaching level of center, and month of hospital admission.

eFigure 5A

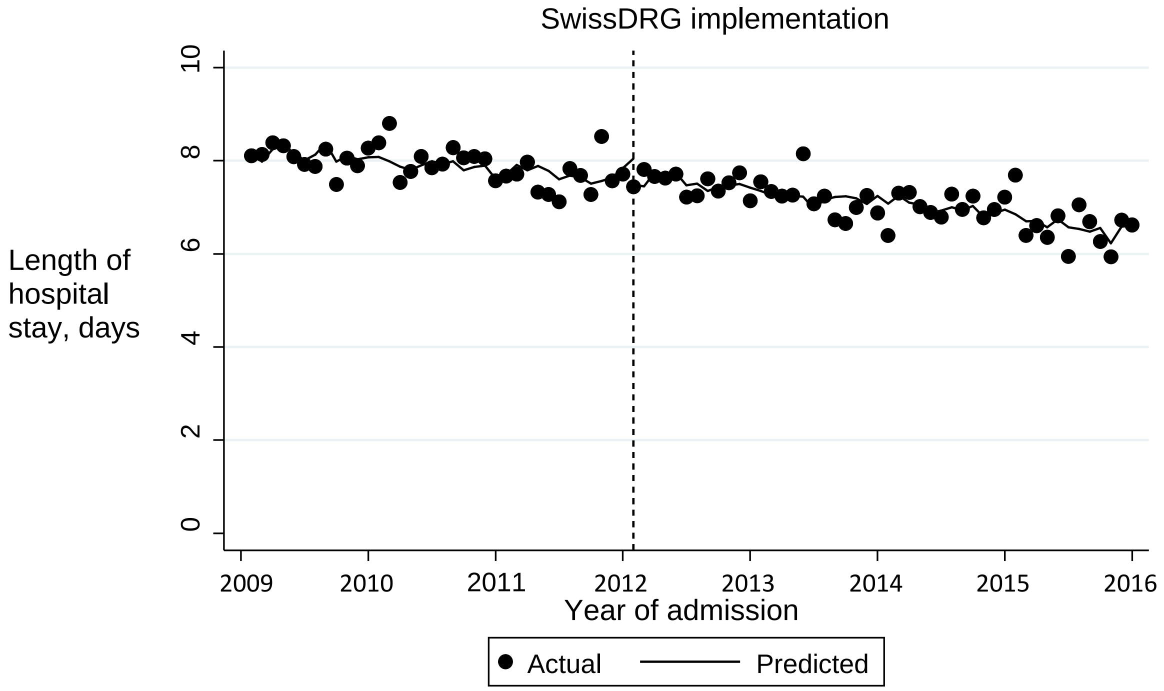

Prais-Winsten and Cochrane-Orcutt regression - lag(1)

©2019 Kutz A et al. *JAMA Network Open.*

eFigure 5B

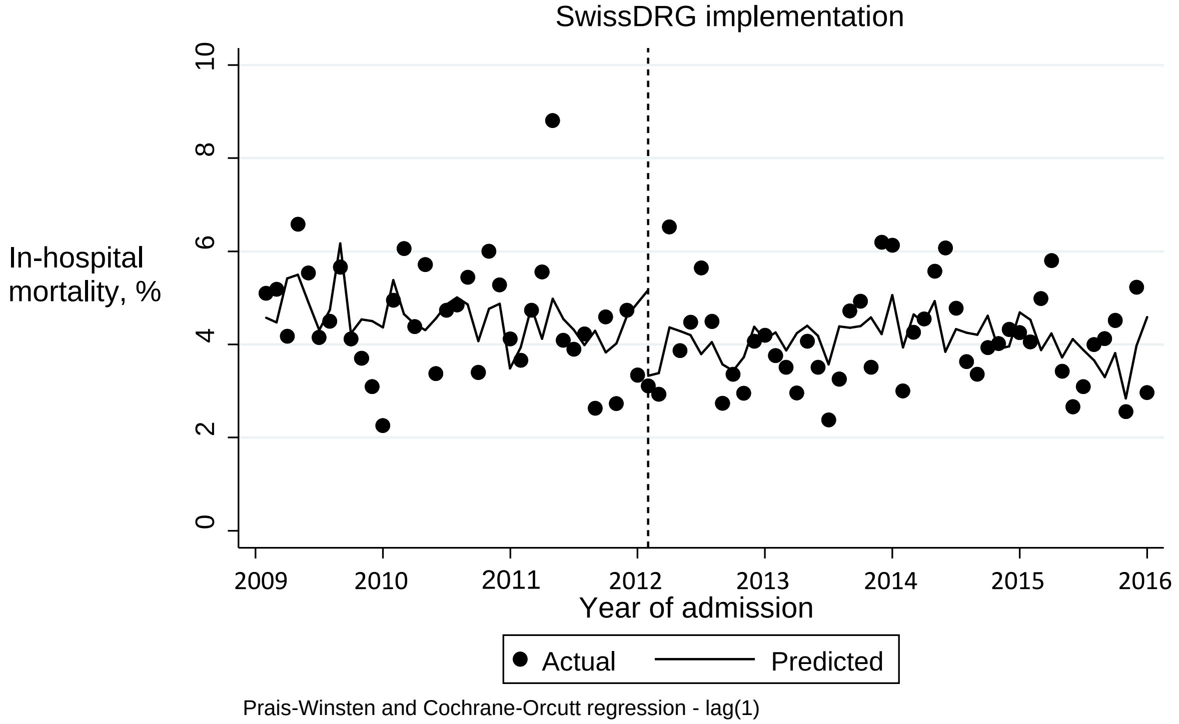

eFigure 5C

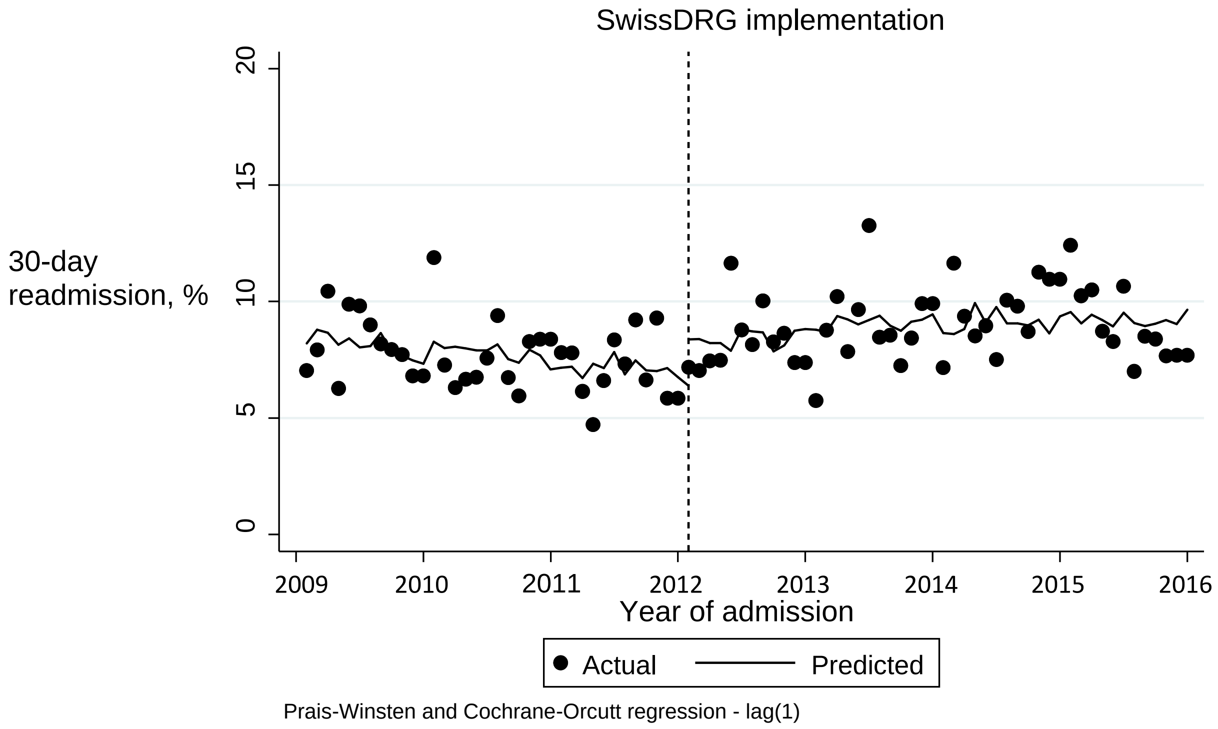

©2019 Kutz A et al. *JAMA Network Open.*
